# CONNECTED YET VULNERABLE: SOCIAL ISOLATION PROFILES AND MENTAL HEALTH AMONG OLDER ADULTS WITH LOW SES

**DOI:** 10.1093/geroni/igae098.2535

**Published:** 2024-12-31

**Authors:** Pildoo Sung, June Lee, Angelique Chan

**Affiliations:** Hanyang University, Seoul, Republic of Korea; Duke-NUS Medical School, Singapore, Singapore; Duke-NUS Medical School, Singapore, Singapore

## Abstract

Despite growing attention to social isolation and loneliness, there remains a gap in understanding how these factors intersect in shaping comprehensive social isolation profiles among vulnerable older adults and their mental health implications. Using various objective and subjective indicators of social isolation, this study aimed to identify social isolation profiles and their direct and stress-buffering roles against depressive symptoms among community-dwelling older adults with low socioeconomic status. Data were collected from 881 older adults aged 55 and above in the Chin Swee area of Singapore where older adults with lower socioeconomic status reside in small rental housing. Latent class analysis and multivariable regression yielded three key findings. First, four distinct social isolation profiles were identified, namely “strongly connected, less lonely (27.9%),” “moderately connected, less lonely (56.4%),” “moderately connected, moderately lonely (11.2%),” and “restricted, severely lonely (4.4%).” Second, older adults in the “moderately connected, moderately lonely” and “weakly connected, severely lonely” profiles tended to report poorer mental health than those in the “strongly connected, less lonely” profile. Third, “moderately connected and moderately lonely” older adults were at a higher risk of depression in the presence of significant life stressors, compared to their less lonely counterparts with strong social connectedness. These findings highlight the need for a comprehensive approach to addressing social isolation and loneliness among disadvantaged older adults in the community. Interventions should not only target disconnected and severely lonely older adults but also extend support to moderately connected yet lonely older adults, recognizing their vulnerability to life stressors.

